# Editorial: Cell Cross-talk in Diabetic Kidney Diseases

**DOI:** 10.3389/fmed.2022.849830

**Published:** 2022-02-23

**Authors:** Quan Hong, Cijiang He, Fan Yi

**Affiliations:** ^1^Department of Nephrology, Chinese PLA General Hospital, Beijing, China; ^2^Mount Sinai Hospital, New York, NY, United States; ^3^Department of Pharmacology, School of Basic Medical Sciences, Shandong University, Jinan, China

**Keywords:** diabetic nephropathy, cell crosstalk, renal fibrosis, diabetic kidney disease, chronic kidney disease

Diabetic kidney disease (DKD) is the leading cause of new-onset end-stage renal disease (ESRD). Although the development of clinical therapy for DKD has made great progress, the progression of DKD still cannot be controlled. Therefore, further study of the pathogenesis of DKD and improvements in DKD treatment are crucial for prognosis. Here, there are evidences suggest the cell crosstalk in the pathogenesis of DKD could provide mechanistic clues that underlie DKD and provide novel avenues for therapeutic intervention.

Liu et al. applied secreted protein comparison and verification experiments indicated that WFDC2 from the tubule could downregulate PEX19 levels at the glomeruli in diabetic kidney disease (DKD). This study revealed the distinctive crosstalk pathways of the tubules and glomeruli and identified interacted genes during kidney disease progression. Feng et al. demonstrated HIF-1α/Notch1 pathway of M1 macrophage could be activated by endothelial cell dysfunction in DKD mouse, and PPAR-α agonist fenofibrate had the protective effect on DKD by reducing M1 macrophage recruitment via inhibiting HIF-1α/Notch1 pathway (Liu et al.). Li Q. et al. uncovered S-nitrosylation of Myo9A, actin, and RhoA as an integrated signaling crosstalk that reversibly transduces metabolic cues to regulate actin dynamics and podocyte motility in DKD (Feng et al.). It suggested that dysregulation of the signal axis may contribute to the pathogenesis of advanced DKD and may be amenable to therapeutic targeting.

During diabetic nephropathy, endothelial cells, and podocytes are stressed and damaged. Besides, each can communicate with the other, directly affecting the progression of glomerular injury. Glomerular ECs are crucial actors of DKD pathophysiology, and cross-communications with podocytes constitute major events for diabetic renal disease progression. Mahtal et al. emphasized new treatments that aim to prevent microvascular injury or restore microvascular function could be an effective strategy for preventing; or even reversing DKD.

Single-cell RNA sequencing (scRNA-seq) technology provided new insight into cellular heterogeneity and genetic susceptibility regarding DKD at cell-specific level. Based on scRNA-seq it is enable a much deeper understanding of cell-specific processes such as interaction between cells. Du et al. highlighted scRNA-seq research on intra- or extra-glomerular cell crosstalk and cellular targets for DKD (Li T. et al.), including crosstalk between podocyte and GEC, podocyte and parietal epithelial cell (PECs), glomerular mesangial cell (GMC), and other glomerular cell types. In addition, Li T. et al. identified a subgroup of glomerular endothelial cells with pro-angiogenesis characteristics in DKD using an online single-cell RNA profile (Wei et al.). Also, immune cells such as macrophages, T lymphocytes, B lymphocytes, and plasma cells contribute to the disease progression. There is a complicated cellular crosstalk inside glomeruli. Dysfunction of glomerular endothelial cells and immature angiogenesis result from the activation of both paracrine and autocrine signals. Based on snRNA-seq data of DKD (He et al.), Wei et al. revealed cell-to-cell interactions via integrin pathways are increased, mesangial cells are stimulated and glomeruli-tubular communication is strongly enhanced in DKD progression. This work found the level of glomerular FGF1 is positively associated with the level of GFR, while the levels of glomerular NRP1, tubular COL4A1, and tubular NRP1 are negatively associated with the level of GFR. This study furthers our understanding of cell cross-talk in DKD and reveals novel mechanisms, new biomarkers, and potential therapeutic targets to benefit patients.

Recent studies have shown that ncRNAs play an important role in the occurrence and development of DKD and participate in the regulation of oxidative stress in DKD. He et al. summarized the functions and mechanisms of ncRNAs in DKD-related oxidative stress (Xu et al.). These ncRNAs would play a pivotal role in the cell crosstalk of DKD progression. Quercetin antagonizes glucose-induced renal injury by suppressing aerobic glycolysis via HIF-1α/miR-210/ISCU/FeS pathway in mesangial cells [9].

In summary, contents of our topic provided valuable insights into cell crosstalk in DKD. Effective strategy for preventing; or even reversing DKD, may consider crosstalk within the glomerular or/and tubular system.

## Author Contributions

QH prepared the draft. CH and FY revised it. All authors contributed to the article and approved the submitted version.

## Conflict of Interest

The authors declare that the research was conducted in the absence of any commercial or financial relationships that could be construed as a potential conflict of interest.

## Publisher's Note

All claims expressed in this article are solely those of the authors and do not necessarily represent those of their affiliated organizations, or those of the publisher, the editors and the reviewers. Any product that may be evaluated in this article, or claim that may be made by its manufacturer, is not guaranteed or endorsed by the publisher.

